# Newborn Screening and Genetic Analysis Identify Six Novel Genetic Variants for Primary Carnitine Deficiency in Ningbo Area, China

**DOI:** 10.3389/fgene.2021.686137

**Published:** 2021-06-24

**Authors:** Xiangchun Yang, Qiong Li, Fei Wang, Lulu Yan, Danyan Zhuang, Haiyan Qiu, Haibo Li, Liang Chen

**Affiliations:** ^1^The Central Laboratory of Birth Defects Prevention and Control, Ningbo Women and Children’s Hospital, Ningbo, China; ^2^Neonatal Screening Center, Ningbo Women and Children’s Hospital, Ningbo, China; ^3^Department of Pediatrics, Ningbo Women and Children’s Hospital, Ningbo, China; ^4^Department of Gynaecology, Ningbo Women and Children’s Hospital, Ningbo, China

**Keywords:** primary carnitine deficiency, newborn screening, free carnitine, novel variants, *SLC22A5*

## Abstract

Primary carnitine deficiency (PCD) is an autosomal recessive disorder that could result in sudden death. It is caused by a defect in the carnitine transporter encoded by *SLC22A5* (Solute Carrier Family 22 Member 5, MIM:603377). Currently, a number of variants in *SLC22A5* have been identified, however, the PCD prevalence and its variants in Ningbo area are unclear. In this study, we screened 265,524 newborns by using tandem mass spectrometry. Variants in *SLC22A5* were further detected by next-generation sequencing in individuals with abnormal free carnitine levels (C0). We identified 53 newborns with abnormal C0 levels and 26 with variants in *SLC22A5.* Among them, 16 with compound heterozygous or homozygous variants in *SLC22A5* were diagnosed with PCD, suggesting the PCD birth prevalence in Ningbo city was 1/16,595. Moreover, the C0 level was significantly (*P* = 0.013) higher in PCD patients than in those with one variant. Besides, the c.1400C > G (p. S467C) and c.51C > G (p. F17L) variants were the most frequent and six novel variants are all predicted to be damaging. This study reports the largest PCD patients in Ningbo area by newborn screening and expands the variant spectrum of *SLC22A5*. Our findings demonstrate the clinical value of combining NBS program results with DNA analysis for the diagnosis of PCD.

## Introduction

Primary carnitine deficiency (PCD, MIM:212140) is an autosomal recessive disorder of carnitine transportation. Its estimated incidence ranges between 1/40,000 and 1/120,000 across countries with different ethnicities (Magoulas and El-Hattab, 2012; Lahrouchi et al., 2017). *SLC22A5* (Solute Carrier Family 22 Member 5, MIM:603377) as the causal gene for PCD spans around 30 kb on chromosome 5q31.1 and encodes organic cation transporter type 2 (OCTIN2) (Nezu et al., 1999; Li et al., 2010; Kilic et al., 2012). OCTN2 operates as a Na^+^ -independent organic cation transporter as well as a high affinity Na^+^ -dependent carnitine transporter (Longo et al., 2016). Defective carnitine transport could result in urinary carnitine wasting, low serum carnitine levels, and decreased intracellular carnitine accumulation (Longo et al., 2006; Longo et al., 2016). When carnitine supplements are not promptly started, patients with PCD can develop an acute metabolic decompensation in their early life, or their later life with skeletal and cardiac myopathy/sudden death from arrhythmia (Wang et al., 2000; Rose et al., 2012; Longo et al., 2016). Hence, early diagnosis and treatment of PCD can prevent metabolic decompensation and death, and the long-term prognosis is favorable.

With the advent of expanded newborn screening (Dhondt, 2010; Lindner et al., 2011; Tiwana et al., 2012; Therrell et al., 2015; Karaceper et al., 2016; Wang et al., 2019), infants with PCD can be identified based on their free carnitine (C0) level. When the level is low, the plasma/urine carnitine, plasma acylcarnitine profile, and urine organic acids in mothers of these infants should be evaluated since the primary or secondary carnitine deficiency could be caused by a maternal disorder (Longo et al., 2016). The diagnosis can be confirmed by verifying reduced carnitine transport activity (<20% of normal controls) in skin fibroblasts from the patient or by mutational analysis of the *SLC22A5* gene.

Here, we performed a large-scale screen on PCD by using MS/MS and DNA sequence analysis in Ningbo area, China. We identified 53 out of 265,524 newborns have low C0 levels. Further genetic analysis identified 16 of them with biallelic heterozygous/homozygous mutations and 10 newborns with one mutation in *SLC22A5*. These 16 newborns were confirmed to have PCD and mothers of the 10 infants with one variant in *SLC22A5* were suspected to have PCD. Infants with maternal PCD were found to have higher C0 levels than those in PCD patients. Besides, six novel variants in *SLC22A5* were identified in this area and all of them were predicted to be damaging.

## Materials and Methods

### Study Population and Patients

A total of 265,524 newborns of Chinese Han ethnicity were screened by MS/MS from July 2014 to March 2019 at the central laboratory of birth defects prevention and control in Ningbo Women and Children’s Hospital. An infant is diagnosed with PCD if the free carnitine level in dried blood spot (DBS) is below the cutoff established in our laboratory (described below), and with compound heterozygous or homozygous variants in *SLC22A5.* The levels of acetylcarnitine and propionylcarnitine were also measured at the same time.

### MS/MS Analysis

For each DBS card, one 3.20 mm diameter disc (equal to 3.20 μL of blood) was punched into U-96-well microplates and 100 μL of an extract solution was added to each well. After incubating for 45 min, 75 μL of the supernatant was transferred into a fresh V-96-well microplate and let to stand for 2 h at room temperature. This final solution was subsequently used for MS/MS analysis (NeoBase^TM^ Non-derivatized MSMS Kit, PerkinElmer, United States). MS/MS analysis was performed using ACQUITY UPLC H-Class XEVO TQD (Waters TQD, United States). Approximately 15 μL of a working solution was directly injected for the analysis. All chromatograms were analyzed with Waters MassLynx v4.1 software (Waters TQD, United States).

### Diagnostic Criteria

The concentration of C0 was measured by MS/MS in the DBS, cutoff values were set as 9.50–57.00 μM, which were adjusted values (considering the probability of missed cases and false negatives on newborn screening) based on 0.5 and 99.5% percentiles among all the samples. Newborns were diagnosed to have PCD based on the following criteria:

(1) Newborns with C0 values < 9.50 μM were rescreened (second screening) 2–3 days after the first screening.
(2) Newborns with persistent low C0 levels (C0 < 9.5 μM) were readmitted for further diagnosis. Pathogenic variants in *SLC22A5* gene were detected for these infants.
(3) Newborns with compound heterozygous or homozygous variants in *SLC22A5* gene were confirmed to have PCD.

For newborns with at least one variant in *SLC22A5*, the C0 level and variants in their mothers were detected. Mothers were diagnosed to be PCD when they have compound heterozygous or homozygous variants in *SLC22A5* and C0 value lower than 9.5 μM.

### Genetic Testing and Bioinformatics Analysis

Genomic DNA was extracted from dried blood spot or peripheral blood obtained from patients and their parents using the OMEGA Genomic DNA Extraction Kit (OMEGA Biotech, United States). Subsequently, targeted sequencing was performed by the basic edition panel of inherited metabolic diseases (Genuine Diagnostic Laboratory, Hangzhou, China) to detect 94 genes, including *SLC22A5, PAH, PTS, MUT*, and other genes. The sequences of target regions were enriched by multiple probe hybridizations using Agilent SureSelect Human Exon Sequence Capture Kit. The capture products were then purified using Agencourt AMPure XP beads (Beckman Coulter). After purification and quality test, the sequencing libraries were quantified by Illumina DNA standard and Primer Premix Kit (kapa), and then massively parallel sequenced by Illumina MiSeq platform. All potentially pathogenic variants were verified by Sanger sequencing using the specific primers. PCR (polymerase chain reaction) conditions were according to TaKaRa LA PCR^TM^ Kit Ver.2.1 (TaKaRa). Trans status of all compound heterozygous variants has been tested.

All identified variants were checked using databases including the Human Gene Mutation (HGMD) Database^1^, ClinVar^2^, ExAC consortium^3^, gnomAD^4^, 1,000 Genome Project database^5^, laboratory internal database (∼20,000 mutations) and literature. The novel missense variants were further assessed for possible pathogenicity based on tools including SIFT, PolyPhen-2, and MutationTaster integrated in VarSome^6^. The variants were classified according to the standards and guidelines issued by the American College of Medical Genetics and Genomics (ACMG) (Richards et al., 2015).

### Treatment and Follow-Up

All patients were defined as asymptomatic after diagnosed with 100 mg/kg/day of L-carnitine orally, three times. The C0 levels of patients were regularly reviewed during treatment (C0 at about 20 μM, a variety of other acylcarnitines in the normal range). Carnitine concentrations including acylcarnitine (C2) and propionylcarnitine (C3), echocardiogram, electrocardiogram, liver function, and creatine kinase were also examined regularly, and patients were followed up at 0.2–4 years old.

### Statistical Analysis

The statistical analysis was performed in R3.6. The difference in metabolite measurements (C0, C2, and C3) between newborns with PCD and newborns with maternal PCD was calculated based on two-tailed *t*-tests. *P* < 0.05 was considered to be statistically significant.

## Results

### Prevalence of PCD in Ningbo Area

**FIGURE 1:**
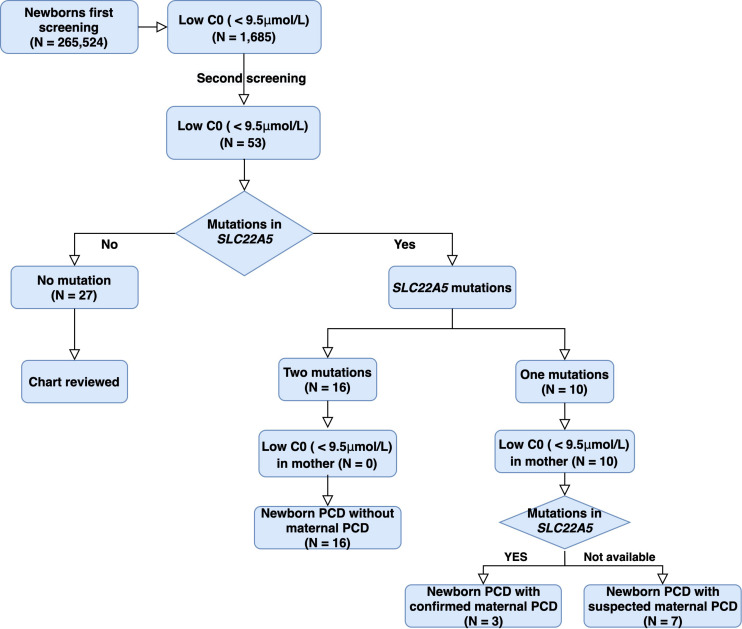
Study design and pipeline of PCD identification by NBS and genetic analysis in Ningbo city. C0: free carnitine; PCD: Primary carnitine deficiency.

A total of 265,524 newborns were screened in Ningbo area during this study. The concentrations of free carnitine (C0) level in DBSs were detected with MS/MS. The levels of free carnitine, acetylcarnitine (C2), and propionylcarnitine (C3) cumulative percentiles in the DBS were analyzed by MS/MS (Table 1). Among them, 241,289 health screening samples collected over 5 years were used as the reference data in our laboratory. We used 0.5 and 99.5% percentiles as the cutoff values for each carnitine concentration. Considering the probability of missed cases and false negatives on newborn screening, the corresponding cutoff values of C0, C2, and C3 in the clinic were then adjusted to be 9.50–57.00, 4.00–48.30, and 0.42–4.50 μM in our laboratory. There were 1,685 newborns identified as positive due to low free carnitine (1 in 158) in the first screening. Of these, 53 subjects were suspected to be PCD with abnormal C0 value in the second screening, giving a positive predictive value of 3.15%. Genetic analysis identified 26 out of 53 newborns with variants in *SLC22A5*, and 16 of them were confirmed to be PCD since they have compound heterozygous or homozygous variants in *SLC22A5*, suggesting the birth prevalence of PCD in Ningbo city was 1/16,595. Ten infants were with one variant in *SLC22A5* and mothers of them were suspected to be maternal PCD considering their low C0 level (C0 < 5μM). Among them, three accepted genetic screening and were all confirmed to have PCD after identification of compound homozygotes in *SLC22A5*, other mothers who disagreed genetic screening were classified as suspected maternal PCD (Figure 1).

**TABLE 1:** Cutoff values and 0.5%/99.5% percentiles used for free carnitine (C0), acetylcarnitine (C2), and propionylcarnitine (C3).

		**Normal population (μM)**					
**Percentile**		**0.5%**		**99.5%**		**Cutoff values**	
**Marker**	***N***	**Value**	95%CI*	**Value**	**95%CI**	**Low value**	**High value**
C0	241289	9.34	(9.28, 9.43)	46.98	(46.16, 47.70)	9.50	57.00
C2	241289	6.12	(5.96, 6.26)	39.16	(38.50, 39.65)	4.00	48.30
C3	241289	0.50	(0.49, 0.51)	3.88	(3.82, 3.94)	0.42	4.50

*CI, confidence interval.

### Clinical and Biochemical Findings

**FIGURE 2:**
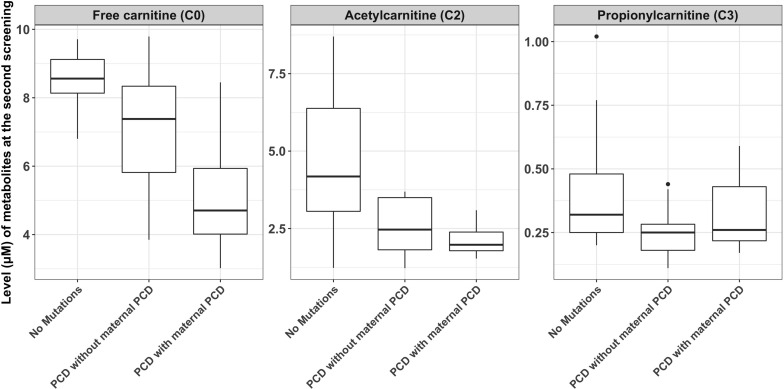
The comparisons of metabolite measurements between newborns with primary carnitine deficiency (PCD), newborns with no mutations in SLC22A5 and newborns with maternal PCD.

C0, C2, and C3 levels were markedly decreased in the 26 newborns with PCD. Among these newborns, the mean concentrations of C0 in infants without maternal PCD and infants with maternal PCD (including confirmed and suspected maternal PCD) were 6.96 ± 1.83 and 5.05 ± 1.67 μM, C2 levels were 2.55 ± 0.89 and 2.15 ± 0.51 μM, and C3 levels were 0.24 ± 0.10 and 0.32 ± 0.14 μM, respectively (Table 2 and Figure 2). Although plasma C0 was reduced in infants with PCD compared to normal newborns, the concentrations of C0 was significantly (*P* = 0.013) higher in patients without maternal PCD than those with maternal PCD. Similar trends were observed for C2 but not in C3. The level of C3 was higher in patients with maternal PCD (Table 2). The concentration of C0 increased to 10–20 μM in newborn PCD after treatment with L-carnitine. We followed up with these patients at 0.2–4 years old. All of the patients were full-term children with normal birth weight, except for Case 2 and Case 26 who were preterm with gestational at age 36/4 and 36/6, respectively.

**TABLE 2:** Levels of free carnitine (C0), acetylcarnitine (C2), and propionylcarnitine (C3) in patients with primary carnitine deficiency (PCD) and maternal PCD.

**Marker (μM)**	**PCD without maternal PCD^a^ (*N* = 16)**	**PCD with maternal PCD (*N* = 10)**	***P*-value**
C0	6.96 ± 0.46^b^	5.05 ± 0.53	0.013
C2	2.55 ± 0.22	2.15 ± 0.16	0.150
C3	0.24 ± 0.024	0.32 ± 0.043	0.104

*^a^Maternal PCD includes confirmed maternal PCD and suspected maternal PCD. ^b^Standard error.*

### Genetic Analysis and Novel Variants

We sequenced 53 newborns with low C0 levels for a definite diagnosis and identified 26 of them with variants in *SLC22A5* (RefSeq NM_003060.4) (Table 3). There are 16 infants with compound heterozygous or homozygous variants and ten infants with one variant. The c.1400C > G (p.S467C) and c.51C > G (p.F17L) variants were most frequent in *SLC22A5* in Ningbo area, with a frequency of 48.84% (21/43) and 16.28% (7/43), respectively. Three of the 16 cases were detected to have homozygous variants (c.1400C > G variant), and other cases were compound heterozygous. The mutated allele in *SLC22A5* for the ten infants with one variant was suspected to be a maternal allele since their mothers were suspected to have PCD (C0 levels of their mothers were lower than 5 μM). Mothers of three newborns (case 24–26) agreed to genetic screening and all of them were confirmed to be maternal PCD since they have homozygous mutations (c.1400C > G variant) in *SLC22A5*. We found six novel variants (c.1161_1162insA, c.1173G > A, c.1343T > G, c.137_159del, c.1420A > C, and c.1490G > A) which have not been reported in the HGMD Database or the *OCTN2* Database at ARUP Laboratories^7^. We assessed the possible pathogenicity of these novel variants based on four bioinformatic tools (SIFT, PolyPhen-2, PROVEAN, and MutationTaster) integrated in VarSome (Kopanos et al., 2019). c.1420A > C was classified to be variant of uncertain significance by ACMG (Supplementary Table 1). Other five mutations were classified as pathogenic or likely pathogenic. Furthermore, all these novel variants were predicted to have “damaging” effects by at least one method (Table 4).

**TABLE 3:** Clinical and genetic characteristics of the *SLC22A5* variants.

**Case**	**Gender**	**Birth weight, KG**	**Gestational weeks**	**C0 level, μM**		***SLC22A5* gene**	
				**Primary screening**	**After treatment**	**Variant allelic 1**	**Variant allelic 2**
**Newborn PCD**							
1	F	3.650	38 + 3	3.85	14.78	c.1195C > T	**c.1420A > C^a^**
2	F	2.800	36 + 4	4.30	24.09	c.51C > G	c.760C > T
3	M	3.150	38 + 0	4.44	17.49	c.760C > T	c.1400C > G
4	F	3.250	38 + 6	4.61	30.81	c.1400C > G	**c.1161_1162insA**
5	F	3.200	37 + 3	6.22	12.61	c.1400C > G	**c.1173G > A**
6	M	3.150	38 + 5	6.34	23.43	c.51C > G	c.1400C > G
7	F	2.750	39 + 6	7.29	17.46	c.51C > G	**c.797C > T**
8	M	3.450	37 + 2	7.06	23.77	c.428C > T	c.1400C > G
9	M	3.500	39 + 3	7.47	15.38	c.1400C > G	c.1400C > G
10	M	3.250	39 + 0	7.78	14.88	c.1400C > G	c.51C > G
11	M	2.700	40 + 1	7.94	21.03	c.51C > G	c.761G > A
12	F	3.800	39 + 1	8.29	23.62	c.680G > A	c.1400C > G
13	M	3.950	40 + 3	8.48	39.14	c.1400C > G	**c.1343T > G**
14	M	3.150	39 + 1	8.49	23.43	c.1400C > G	c.1400C > G
15	M	3.250	38 + 0	9.34	19.26	c.1400C > G	**c.1490G > A**
16	F	2.800	37 + 4	9.49	21.12	c.1400C > G	c.1400C > G
**Infants with suspected maternal PCD**							
17	M	3.200	38 + 2	3.02		c.51C > G	
18	M	3.550	40 + 3	6.48		c.1195C > T	
19	M	2.800	38 + 5	4.56		c.1400C > G	
20	F	3.400	39 + 2	3.07		**c.137_159del**	
21	F	3.750	40 + 1	6.03		c.1400C > G	
22	M	3.750	39 + 1	4.85		c.51C > G	
23	M	3.950	38 + 2	5.65		c.1400C > G	
**Infants with confirmed maternal PCD**							
24	M	3.200	39 + 0	8.45		c.1400C > G	
25	M	3.200	39 + 0	4.54		c.1400C > G	
26	F	3.400	36 + 6	3.84		c.1400C > G	

*^*a*^Black bold in the table indicates novel variants identified in this study.*

**TABLE 4:** Analysis and *in silico* prediction of the novel *SLC22A5* gene variants.

**No.**	**Location^a^**	**Nucleotide change**	**Protein change**	**SIFT^b^**	**PolyPhen-2^b^**	**PROVEAN^b^**	**Mutation taster^b^**
**1**	Exon 8	c.1420A > C	p. Ser474Arg	D	D	D	D
**4**	Exon 7	c.1161_1162insA	p. Val388Serfs*135	N/A	N/A	N/A	D
**5**	Exon 7	c.1173G > A	p. Trp391*	N/A	N/A	N/A	D
**13**	Exon 8	c.1343T > G	p. Val448Gly	D	B	D	D
**15**	Exon 9	c.1490G > A	p. Ser497Asn	D	B	N	D
**20**	Exon 1	c.137_159del	p. Pro46Argfs*84	N/A	N/A	N/A	D

*^*a*^The reference sequence used in this study was based on the NCBI37/hg19 assembly of the human genome. NM_003060.4 was employed as reference sequence for SLC22A5.*

*^*b*^N/A, not available; B, benign; D, damaging; N, possibly polymorphism; P, probably damaging.*

## Discussion

In this study, we screened 265,524 newborns to detect PCD in Ningbo area, China. In total, 53 out of 265,524 newborns were suspected to have PCD considering their low level of free carnitine (C0) measured by MS/MS. Genetic analysis confirmed 16 of them to be PCD patients (birth prevalence = 1/16,595) since they have compound heterozygous or homozygous variants in *SLC22A5*. In addition to the common variants, six novel variants in *SLC22A5* were found in the PCD patients in this area and all of them were predicted to be damaging.

The incidence of PCD ranges from 1:40,000 to 1:120,000 in different countries or regions: with 1:20,000–1:70,000 in the United States (Gallant et al., 2012), 1:40,000 in Japan (Shibata et al., 2018) and 1:120,000 in Australia (Wiley et al., 1999; Webster et al., 2003), and the incidence of heterozygotes for PCD in the population is 0.5–1%. The highest incidence of PCD is in the Faroe Islands, where the prevalence is 1:300. In our study, 16 patients (16/53, 30.19%) with PCD were diagnosed by biochemical and molecular analysis, and the prevalence of PCD was approximately 1:16,595 in Ningbo area. The incidence was a bit higher than that in the study from Zhejiang Province (∼1:30,182), China, in which Ningbo area was excluded (Zheng et al., 2017; Lin et al., 2020). All these results suggest PCD incidence rate is different across different countries and east Asians are more likely to develop PCD than individuals with European ancestry.

This study used a large number of samples to analyze the reference interval of each acylcarnitine, which improves the screening and diagnosing accuracy of PCD in Ningbo area. The cutoff C0 values used in our laboratory (9.50–57.00 μM) were lower than the previously reported values (15.0–95.0 μM) in the children’s hospital, Zhejiang university school of medicine (Huang et al., 2011). The newborn screening data explored many genetic disorders across a wide spectrum of racial/ethnicity classifications, different cutoff values might be required to reduce the risk of false-positive cases in different laboratories or regions. The difference of C0, C2, and C3 concentration between newborns with PCD and maternal PCD is rarely reported based on our knowledge. As the results shown in Table 2 and Figure 2, newborns with PCD were metabolically dissimilar from those with maternal PCD. We found that the mean initial free carnitine (C0) concentrations were significantly lower (*P* = 0.013) in infants with maternal PCD (5.05 ± 1.67 μM) than those with PCD (6.96 ± 1.83 μM). This suggests using the C0 level to determine PCD can have false positives and next-generation sequencing can improve the diagnosis precision. However, the difference in C2/C3 levels between these two groups did not show a significant difference. Importantly, we found that the concentration of C0, C2, and C3 can be increased to normal carnitine levels after regular systemic treatment with oral levocarnitine (L-carnitine) (Table 3). Patients with infantile metabolic disturbance and childhood myopathy will have a good prognosis. The treatment method is simple, safe, and effective.

More than 110 variants have been reported in the *SLC22A5* gene region and they were found to be associated with PCD. Among them, c.1400C > G (p.S467C) is considered to be the most common variant in Chinese patients. In our study, the most frequent variants were c.1400C > G (48.84%) and c.51C > G (16.28%) in Ningbo area, which were similar to other places in China. There were 15 different variants in *SLC22A5* gene identified in our study and six of them are novel variants (c.1161_1162insA, c.1173G > A, c.1343T > G, c.137_159del, c.1420A > C, and c.1490G > A). c.1490G > A was predicted to be probably damaging. c.1173G > A variant was considered as early termination and predicted to be damaging. The c.137_159del (p. Pro46Argfs^∗^84) is a frameshift variant with truncated protein, c.1161_1162insA is also a frameshift variant and protein features might be affected. According to the UniProt database analysis, the protein position at 88-517 of *SLC22A5* (*UniProt ID: O76082*) is a major facilitator superfamily domain, the novel variants except c.137_159del are all included in the domain and these variants may affect structure and function resulting in PCD. Further protein function analysis would be required to validate the predicted functions of these novel variants.

## Conclusion

In conclusion, 53 out of 265,524 newborns in Ningbo area were identified to have low free carnitine (C0) level and further genetic analysis found 26 of them carrying *SLC22A5* variants. Among them, 16 newborns were confirmed to have PCD, and three have maternal PCD. c.1400C > G (p. S467C) and c.51C > G (p. F17L) were the most frequent variants in the *SLC22A5* gene in Ningbo area and six novel variants were discovered in this study. These new variants expand the variant spectrum of *SLC22A5* and provide additional molecular evidence for the etiological diagnosis of the patient with PCD.

## Data Availability Statement

The data presented in the study are deposited in the European Nucleotide Archive (ENA) repository, accession number: PRJEB44918.

## Ethics Statement

The studies involving human participants were reviewed and approved by the Ethics Committee of the Ningbo Women and Children’s Hospital. Written informed consent to participate in this study was provided by the participants’ legal guardian/next of kin.

## Author Contributions

All authors listed have made a substantial, direct and intellectual contribution to the work, and approved it for publication.

## Conflict of Interest

The authors declare that the research was conducted in the absence of any commercial or financial relationships that could be construed as a potential conflict of interest.

## Footnotes

1 http://www.hgmd.cf.ac.uk/ac/index.php

2 https://www.ncbi.nlm.nih.gov/clinvar/

3 http://exac.broadinstitute.org/

4 https://gnomad.broadinstitute.org/

5 http://www.1000genomes.org/

6 https://varsome.com

7 http://www.arup.utah.edu/database/OCTN2/OCTN2_display.php
