# Supplementary material for: Newborn Screening and Genetic Analysis Identify Six Novel Genetic Variants for Primary Carnitine Deficiency in Ningbo Area, China
Source: Front Genet. 2021 Jun 24;12:686137. doi: 10.3389/fgene.2021.686137 (PMC8264545; doi:10.3389/fgene.2021.686137)
Supplement: Supplementary file 1 [file Table_1.DOCX]

**Supplementary Table 1** ACMG classification of the novel *SLC22A5* gene variants.

| No. | Location^1^ | Nucleotide change | ACMG classification | ACMG criteria rules ^2^ | SIFT ^3^ | PolyPhen-2 ^4^ | PROVEAN ^5^ | Mutation Taster ^6^ |
| --- | --- | --- | --- | --- | --- | --- | --- | --- |
| 1 | Exon 8 | c.1420A>C | Uncertain Significance | PM1+PM2+PP3 | 0.001 | 0.93 | -4.2 | 1 |
| 4 | Exon 7 | c.1161_1162insA | Likely Pathogenic | PVS1+PM2 | N/A | N/A | N/A | 0.01 |
| 5 | Exon 7 | c.1173G>A | Pathogenic | PVS1+PM2+PP3 | N/A | N/A | N/A | 1 |
| 13 | Exon 8 | c.1343T>G | Likely Pathogenic | PM1+PM2+PM5+PP3 | 0.001 | 0.289 | -5.25 | 1 |
| 15 | Exon 9 | c.1490G>A | Likely Pathogenic | PM1+PM2+PM3+PP3 | 0.027 | 0.151 | -1.97 | 0.9999 |
| 20 | Exon 1 | c.137_159del | Pathogenic | PVS1+PM2+PP3 | N/A | N/A | N/A | 0.19 |

^1^ The reference sequence used in this study was based on the NCBI37/hg19 assembly of the human genome. NM_003060.4 was employed as reference sequence for *SLC22A5*.

^2^ These rules are mainly from VarSome (<https://varsome.com>). A few adjustments have been made based on knowledge.

^3^ Pathogenicity scores from SIFT. The score ranges from 0.0 (deleterious) to 1.0 (tolerated). N/A means not available.

^4^ This score predicts the possible impact of an amino acid substitution on the structure and function of a human protein, it ranges from 0.0 (tolerated) to 1.0 (deleterious). N/A means not available.

^5^ Variants with scores lower than -2.5 were defined to have a “deleterious” effect. N/A means not available.

^6^ The probability of Mutation taster prediction with values close to 1 corresponding to the most “secure” predictions. N/A means not available.
